# Untargeted Metabolomics of Rind Essential Oils Allowed to Differentiate Two Closely Related Clementine Varieties

**DOI:** 10.3390/plants10091789

**Published:** 2021-08-27

**Authors:** María del Carmen González-Mas, José L. Rambla, Aurelio Gómez-Cadenas, María Amparo Blázquez, María Pilar López-Gresa, Antonio Granell

**Affiliations:** 1 Fundación AgroAlimed (Instituto Valenciano de Investigaciones Agrarias, IVIA), Carretera Moncada-Náquera, Km 4.5, 46113 Moncada, València, Spain; 2 Departamento de Ciencias Agrarias y del Medio Natural, Universitat Jaume I, Av. Vicent Sos Baynat s/n, 12071 Castellón de la Plana, Castelló, Spain; jorambla@uji.es (J.L.R.); cadenas@uji.es (A.G.-C.); 3 Departament de Farmacologia, Facultat de Farmàcia, Universitat de València, Av. Vicent Andrés Estellés s/n, 46110 Burjassot, València, Spain; Amparo.Blazquez@uv.es; 4 Instituto de Biología Molecular y Celular de Plantas, CSIC—Universitat Politècnica de València-Consejo Superior de Investigaciones Científicas, Ingeniero Fausto Elio, 46022 València, València, Spain; mplopez@ceqa.upv.es (M.P.L.-G.); agranell@ibmcp.upv.es (A.G.)

**Keywords:** *Citrus clementina*, Clemenules, Clemenpons, flavedo, volatile organic compounds, antioxidant activity

## Abstract

Chemical characterization of clementine varieties (*Citrus clementina* Hort. ex Tan.) essential oils (EO) can lead to variety identification and valorization of their potential use in food and aroma industries. The goal of this study was the chemometric discrimination between two very closely related and morphologically identical clementine varieties, Clemenules (NL) and Clemenpons (PO), based on their rind EO, to identify the differential volatile organic compounds (VOCs) and to determine their antioxidant capacity. EO rind volatile profile was determined by gas chromatography coupled to mass spectrometry in *Citrus* fruit at different ripening stages grown two independent years in two different locations. Untargeted metabolomics and multivariate data analysis showed an evolution of EO volatile profiles markedly parallel in both varieties. Although EO qualitative composition was identical in both varieties, PLS-DA allowed the identification of characteristic VOCs, quantitatively discriminating them along all the ripening process. PO showed higher accumulation of several mono- and sesquiterpene compounds such as *trans*-carveol, while NL showed higher levels of aldehyde and alcohol non-terpenoids like dodecanal. Both varieties evinced identical EO antioxidant activities, indicating a similar value for food preservation. Hence, untargeted metabolomics approach based on rind EO volatiles was revealed as a powerful technique able to differentiate between morphologically undistinguishable *Citrus* varieties.

## 1. Introduction

Clementine (*Citrus clementina* Hort. ex Tan.) is a *Citrus* fruit originated from the hybridization between mandarin (*C. reticulata* Bl.) and sweet orange (*C. sinensis* Osb.) [1]. This species is composed of several dozens of morphologically close varieties, whose unequivocal identification is complex. Since the chemical variability of *Citrus* essential oils (EO) depends in part on the genetic factors, flavedo EO chemical characterization has been used to variety identification [2,3,4]. Nevertheless, considering that environmental conditions have also a role in the EO profile, only *Citrus* EO of species and varieties grown under similar conditions can be readily compared [5].

The volatile profile of mandarin rind EO is qualitatively similar to that of oranges [6,7]. As in all *Citrus* EO, the most abundant compounds in *C. reticulata* and *C. sinensis* EO are monoterpene compounds, with the hydrocarbon limonene being the major compound in flavedo oil in both species, often representing about 94–97% of the total volatile fraction, although occasionally decreasing down to 60% in both species [7]. Other abundant monoterpenes of both flavedo EO are also hydrocarbons such as γ-terpinene, which sometimes reaches values above 20% in *C. reticulata* [5], and β-myrcene, α-pinene, and β-pinene (from 7% to traces, generally considering trace values those below 0.05%) [7]. Oxygenated monoterpenes are also present in *C. reticulata*, *C. sinensis,* and other *Citrus* EO, such as the alcohols linalool, geraniol, terpinen-4-ol, or *trans*-carveol, the aldehydes citronellal and neral, the ketone carvone, or the esters citronellyl-, neryl-, and geranyl- acetates, with an abundance below 1% with the only exception of linalool, which can reach up to 3–4 % in *C. reticulata* or even more in *C. reticulata* variety ‘Dahongpao’ [7,8,9]. Sesquiterpene compounds have also been reported in *C. reticulata* and *C. sinensis* flavedo EO, both hydrocarbon compounds such as α-copaene or α-humulene and oxygenated like β-sinensal, with values that usually do not exceed 0.1% each [7]. Qualitatively, the main difference of *C. reticulata* EO from other *Citrus* species including *C. sinensis* is the presence of almost exclusive aldehyde compounds derived from the degradation of fatty acids, such as (*Z*)-4-decenal or (*E*)-2-nonenal. However, these compounds, which have also been reported in *C. clementina*, do not usually reach an abundance higher than 0.1% in *C. reticulata* [7].

Numerous biological activities, such as antimicrobial and antioxidant, have been described for *Citrus* EO, being frequently used for this reason in the food industry [8,9,10,11,12,13]. They are also widely used in perfumery due to their attractive smell. Moreover, it has been demonstrated that *Citrus* EO can be effective in controlling and repelling some insect pests [14]. Regarding their physiological function, EO are involved in defense against biotic and abiotic stress and in the attraction of pollinating insects and seed dispersers [15].

Clemenules (NL) is by far the most cultivated clementine variety in Spain, due to its outstanding fruit quality. Clemenpons (PO) appeared in the Valencian region (Spain) as a spontaneous mutation from NL. Their fruits are morphologically indistinguishable, being identical in size, weight, diameter/height ratio, rind size, color, percentage of juice, and absence of seeds. Their harvest period is also very analogous (October–December), although in PO it usually starts and ends a little earlier (2 weeks before) [16]. However, despite their similarity, farmers claim that PO rinds give off an aniseed odor that is not perceived from NL rinds, a trait that could be explained by differences in their flavedo EO composition. This paper focuses on the comparison of the volatile organic compounds (VOCs) in flavedo EO from these two highly related clementine varieties.

In a previous study, we were able to differentiate NL and PO flavedo EO composition from those of other clementines like Arrufatina and Clemensol [17]. This research was done in a single season and location, and it did not allow discrimination between NL and PO varieties. So far, no research has been able to distinguish the flavedo VOCs’ EO of these two varieties. Garcia-Sanchez et al. (2016) could discriminate the VOCs’ juice of eight clementines and one satsuma, including NL and PO varieties [18]. However, the volatile profile in *Citrus* juice is different to that in the rind [19].

The objective of this study was to establish whether a more powerful metabolomic approach would be able to discriminate between NL and PO based on their rind EO, and, if so, to identify the VOCs responsible for that difference. Moreover, the antioxidant activity of NL and PO flavedo EO was also analyzed to determine if there exist differences between them.

## 2. Results and Discussion

Untargeted metabolomics based on gas chromatography-mass spectrometry (GC-MS) and multivariate data analysis (MVDA) of NL and PO flavedo EO from two consecutive seasons and cultivated in two different locations, plot number 5 (P5) and Germplasm Collection plot (GCP), were carried out to determine whether the almost identical varieties, NL and its mutant PO, can be differentiated based on their EO volatile profile. Fruits from both varieties were collected on a weekly basis along 2 months, which was the time the ripe fruit is in good shape and is collected for commercialization.

The mass spectrum profile in the rind EO of both varieties was made up of over 11,000 mass signals corresponding to several hundreds of VOCs. The volatile profile was largely in accordance with those previously reported in *C. clementina* EO [3,4,7,20,21]. In both varieties all along the 2-month harvest time, the most abundant compound was the hydrocarbon monoterpene limonene (Figure S1). A vast majority of the other abundant VOCs in the chromatograms of both varieties were monoterpenes and sesquiterpenes. Most of them were hydrocarbon terpenes such as the monoterpenes α-pinene, β-myrcene, α-phellandrene, and γ-terpinene or the sesquiterpene α-copaene. Nevertheless, some oxygenated terpenes, including monoterpenoids such as linalool, α-terpineol, terpinen-4-ol, *trans*-carveol and carvone, or sesquiterpenoids such as β-sinensal, were also among the most abundant compounds. In addition, some non-terpenoid linear aldehydes such as octanal, decanal, or dodecanal were also rather abundant. The qualitative composition was the same in both varieties.

### 2.1. Principal Component Analysis (PCA)

**Figure 1:**
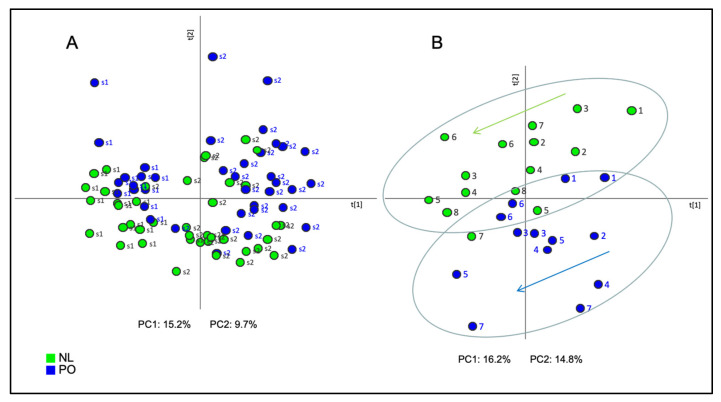
Score plot of the Principal Component Analysis (PCA) based on the whole array of the mass spectra within a *m/z* range from 35 to 220 obtained from samples of Clemenules (NL: green) and Clemenpons (PO: blue) collected during (**A**) both season 1 (s1) and season 2 (s2); (**B**) during s1; numbers 1 to 7 account for harvest day, from earlier (1) to later date (7). Only for NL variety, the later date in s1 was date (8). PC1: first principal component; PC2: second principal component.

As a first approach of the statistical analysis, Principal Component Analysis (PCA) was performed in order to group all samples according to the mass signals of each variety, season, or location. A clear separation between samples collected in season 1 (s1) and season 2 (s2) was observed in the PCA score plot (Figure 1A). The first principal component (PC1), accounting for 15.2% of total variance, explained the changes in the EO chemical composition between the two seasons handled in this study, thus indicating that the environmental effect associated to the different climate conditions between each season had a higher effect on the rind EO than the effects of location or variety. It can also be observed that, within each year, NL and PO tend to separate from each other, meaning that there is some effect due to the variety, although this effect is minor in comparison with the variance due to the season.

When a PCA for each season was performed separately, an evident distinction was shown between both varieties collected during s1 by the second principal component (PC2), explaining 14.3% of data variance, whose samples correspond to a single location (P5) (Figure 1B). However, no clear separation was found to distinguish both varieties collected during s2, which included data from two different locations (P5 and GCP) (Figure S2). Therefore, a more powerful statistical tool was required to unravel consistent differences in the EO volatile composition between NL and PO.

### 2.2. Evolution of the Volatile Profile along Harvest Time

In general terms, the volatile composition of EO was rather stable during the 2-month collecting season in both varieties. Nevertheless, some significant changes were detected in the volatile profile along the 2-month harvest time. When focusing on sampling date, PCA revealed a markedly parallel evolution of the volatile profile in NL and PO along this period (Figure 1B and Figure S2). Analyzing PC1 of both loading plots, EO at the earliest dates was characterized by higher accumulation of several mono- and sesquiterpene hydrocarbons, such as β-phellandrene, β-myrcene, β-pinene, and γ-terpinene (Table S1). These compounds reduced progressively their levels down to 0.6–0.8-fold in the late harvest dates. Nevertheless, no differences were detected for most compounds including limonene. The lower accumulation of several monoterpene hydrocarbons at the end of harvest time had also been reported in other *Citrus* EO, like *C. reticulata*, *C. aurantium* and *C. limon* [8,22,23]. Some of these studies reported a limonene decrease in EO at the end of harvest time [8,23], which was not observed in this study. The decrease in the abundance of monoterpene hydrocarbons along *Citrus* ripening could be explained by the high unsaturation level of these compounds that would make them more unstable to factors such as light or chemical oxidation [23]. In addition, Wang and Liu (2014) reported in *C. reticulata* an increase of sesquiterpene hydrocarbons at the end of the harvesting period [8], although this effect was not observed in our study.

On the other hand, a characteristic feature of late harvest date was the increased accumulation of a set of medium-to-long chain (C_10_-C_18_) double-bonded fatty acid derivatives, which showed a moderate (1.6- to 1.8-fold) but highly significant (*p* < 0.01) increase during harvest time, thus suggesting an enhanced activity of fatty acid metabolism (Table S1). As far as we know, this has never been described in any previous study on the evolution of *Citrus* rind EO composition. These results reveal that there is a mild effect of the harvesting period on the composition of *Citrus* rind EO. It is important to note that the waxes of fruit cuticle are complex mixtures of long-chain lipid compounds including fatty acids, alkanes, or alkenes. The cuticle represents a mechanically resistant barrier with a very important structural function in organ integrity and protection against internal or external stresses, such as cellular turgor pressure, dehydration, or low temperature. As was recently established, wax components seem to also act as signal molecules in some developmental processes related to cell growth and also in defense mechanisms, which could explain the increased level of fatty acid derivatives in EO associated with the fruit ripening [24]. In fact, Wang et al. (2016) showed that wax synthesis is synchronous with fruit maturation in *C. sinensis* and that the genes involved in the biosynthesis of wax are significantly induced at later fruit stages [25]. 

### 2.3. Projection to Latent Structures-Discriminant Analysis (PLS-DA) 

**Figure 2:**
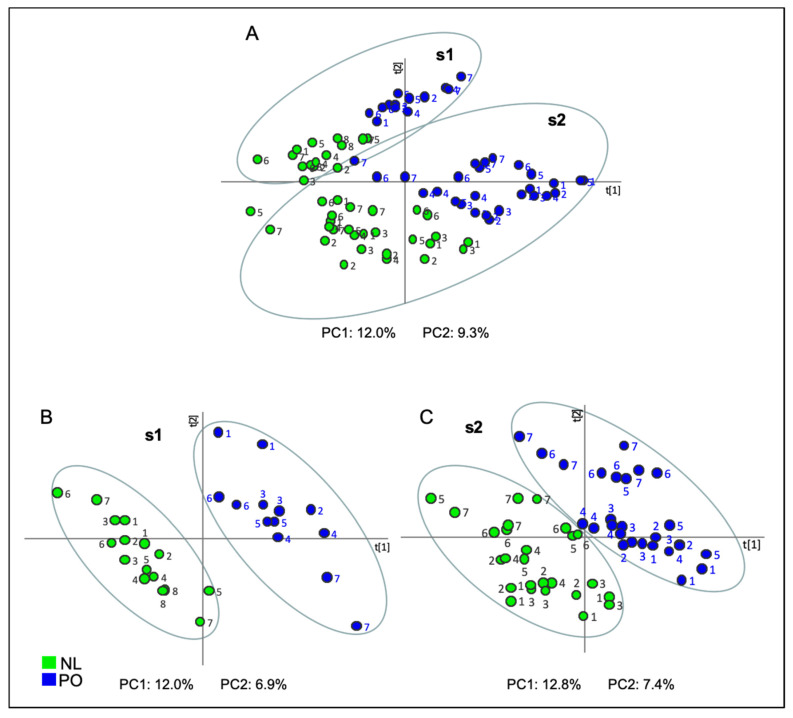
Score plot of Projection to Latent Structures-Discriminant Analysis (PLS-DA) based on the whole array of the mass spectra within a *m/z* range from 35 to 220 obtained from samples of Clemenules (NL: green) and Clemenpons (PO: blue) collected during (**A**) both s1 and s2; (**B**) during s1 in a unique location; (**C**) during s2 in two locations. Numbers 1 to 7 account for harvest day, from earlier (1) to later dates (7). Only for NL variety, the later date in s1 was date (8). PC1: first principal component; PC2: second principal component.

In order to obtain a statistical analysis that separated varieties collected in both seasons and locations, a PLS-DA was performed. Two discrete classes, NL and PO, were created for this supervised MVDA. A complete separation between varieties of both seasons was not achieved by the PLS-DA, although a clear tendency was observed (Figure 2A).

However, when PLS-DA was carried out using the data set of each year independently, a clear differentiation by their content in volatile compounds between both types of varieties was obtained (Figure 2B,C). On the one hand, PLS-DA of data obtained from s1 samples (Figure 2B) showed that PC1, which explained 12.0% of data variance, combined with PC2, which explained 6.9% of data variance, allowed us to perfectly distinguish between both varieties. In this study, both components were somewhat rotated.

On the other hand, the PLS-DA performed using data from samples collected in s2 (Figure 2C), both those grown in P5 and in GCP, perfectly separated both varieties through the combination of PC1, which explained 12.8% of data variance, with PC2, which explained 7.4% of data variance. As in the previous analysis, these components were also rotated.

### 2.4. Identification of Differential VOCs between NL and PO Essential Oil

Analyzing the loading plot of PLS-DA component 1 from all samples collected, both in s1 (in P5; Figure 2B) and in s2 (in P5 and GCP; Figure 2C), a variable importance (VIP) score was generated based on its ability to explain the separation between NL and PO EO, in spite of qualitative similarity of both volatile profiles. This VIP score was defined by a group of VOCs more abundant in one variety than in the other, independently of season, harvest time, or location. These differential VOCs were identified and relatively quantified by calculating the abundance ratio between varieties. Only those VOCs statistically different consistently in both seasons were considered as characteristic of each variety. 

In this manner, Table 1 shows the VOCs of VIP score that were statistically in higher concentration in PO than in NL EO, indicating the abundance ratio between the PO and NL varieties.

**Figure 3:**
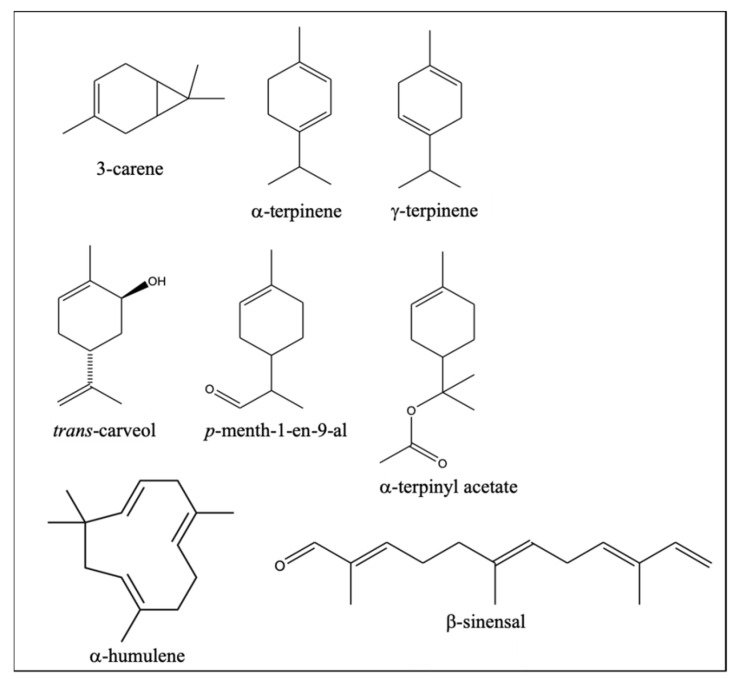
Identified monoterpenes and sesquiterpenes statistically more abundant in Clemenpons EO than in Clemenules EO, according to Table 1 (*p* < 0.05, according to Student’s *t*-test, for each compound).

Interestingly, all the differential VOCs more abundant in PO were terpenoids, mostly monoterpene compounds with 1-*p*-menthene structure (Figure 3). These results seem to indicate that part of terpene biosynthesis pathways is more potentiated in the PO variety than in NL. Some of them were unequivocally identified based on comparison of both mass spectra and retention time with those of pure standards. When standards were not available, similarity of their mass spectra with those in mass spectral libraries allowed us to determine some features about their chemical identity, such as if these VOCs were mono- or sesquiterpenes, or if they were oxygenated (Table 1). In this way, 10 of these differential VOCs resulted to be hydrocarbon and oxygenated monoterpenes and were present in PO in an abundance between 1.3- and 3.3-fold higher than in NL (Table 1). Specifically, three of these compounds were monoterpene hydrocarbons (3-carene, α-terpinene, and γ-terpinene), while the other seven were oxygenated monoterpenes, two of which were unambiguously identified (*trans*-carveol and α-terpinyl acetate), and a third compound, *p*-menth-1-en-9-al, was only tentatively identified by its mass spectrum, since a commercial standard is not available (Figure 3). The remaining four oxygenated monoterpenes could not be reliably identified. Three of the differential oxygenated monoterpenes doubled their abundance in PO compared to NL, like *trans*-carveol, and the other was found to triplicate its abundance, while the differences in monoterpene hydrocarbons were smaller (Table 1).

The monocyclic monoterpene hydrocarbons α-terpinene and γ-terpinene are usually identified in *Citrus* EO (Figure 3). The first is reported in *C. reticulata* in concentrations not higher than 0.3%, whereas in *C. sinensis* it can be present in concentrations close to 1.7% [7,27,28]. In contrast, the latter has been detected in *C. reticulata* even at concentrations almost as high as 20% [5], or at concentrations close to 10% in *C. sinensis* [29]. The bicyclic monoterpene hydrocarbon 3-carene (Figure 3), also more abundant in PO, has less frequently been identified in *Citrus* EO and, when reported, it was always at concentrations below 0.1% in *C. clementina* [2,7,21,30]. The compounds α-terpinene and 3-carene provide to EO a typical lemon odor, whereas γ-terpinene provides a turpentine odor, according to the Flavornet database (Table 1) [26].

As for the two oxygenated monocyclic monoterpenes more abundant in PO unequivocally identified, α-terpinyl acetate and *trans*-carveol (Figure 3), they were previously identified in *C. clementina* EO as traces, in concentrations lower than 0.05% [2,31]. Each of these VOCs provide a different aroma to those of the three monoterpene hydrocarbons mentioned above (Table 1). Specifically, aroma of wax and caraway (*Carum carvi*) are provided by α-terpinyl acetate and *trans*-carveol, respectively. Caraway is an aromatic plant usually used as a food condiment with an anise-like aroma due to the EO composition of their fruits, rich in carvone (44.5–95.9%) and with other terpenes such as *trans*-carveol (0–0.2%) and *trans*-dihydrocarvone (0–0.5%) [26,32,33]. This caraway aroma of *trans*-carveol, together with its double abundance in PO versus NL (Table 1), might explain the particular aroma of PO fruit peel reported by farmers. Nevertheless, it cannot be discarded that some of the other unidentified oxygenated monoterpenes more abundant in PO could also be responsible for the aniseed aroma attributed to PO peel.

The other five differential VOCs of PO were sesquiterpenes (Table 1). Four of them were hydrocarbon, with α-humulene being the only one unambiguously identified. The other compound was an oxygenated sesquiterpene tentatively identified as the aldehyde β-sinensal (Figure 3). Both α-humulene and β-sinensal have been frequently detected in *C. clementina* peel EO, although α-humulene was reported at trace levels and β-sinensal in concentrations reaching 0.3% [2,30]. The monocyclic compound α-humulene provides a wood aroma, whereas the lineal β-sinensal has a sweet aroma [26]. None of the five differential sesquiterpenes doubled their levels in PO with respect to NL, thus suggesting a lower influence on the aroma differentiation between these varieties.

Comparing our results with those reported in clementine juice [18], some similarities can be observed. In that study about VOCs of several juices, it was also possible to distinguish PO from other clementine varieties. One of the differential compounds was the monoterpene carvone, since the PO juice showed the highest content of this volatile. Interestingly, carvone is closely related to *trans*-carveol, one of compounds significantly more abundant in PO than in NL in our study, with both compounds being derived from limonene autooxidation [34,35]. In the case of the fruit of caraway, it has been demonstrated that limonene is transformed into *trans*-carveol by means of the limonene-6-hydrolase enzyme, which is subsequently oxidized by a dehydrogenase to produce carvone [32]. It seems feasible that a similar mechanism may be behind the enhanced *trans*-carveol biosynthesis in PO.

On the other hand, the five VOCs that were part of the VIP score and were significantly more abundant in NL than in PO EO are shown in Table 2. All of them are aliphatic non-terpenoid compounds of eight or more carbon atoms in their chemical structure. Their abundance in PO ranged between 9% and 39% lower than in NL, approximately (Table 2).

According to these results, NL was revealed to show slightly higher accumulation of linear compounds originated from fatty acid oxidation than PO (Table 2). Thus, fatty acid metabolism seems to be more active in NL than in PO fruit rinds. Among these fatty acid derivatives more abundant in NL, the alcohols 1-octanol and 1-decanol provide mossy and fatty aroma, respectively [26]. Both compounds have been often identified in *Citrus* rind EO, especially the first one in *C. sinensis* and *C. reticulata*, with concentrations lower than 0.4% and 0.1%, respectively [2,7,31]. The aldehydes dodecanal and tetradecanal also provide fatty aroma [36,38], and they have been often identified in Citrus rind EO, particularly dodecanal, with a concentration lower than 0.2% [2,7,20,27,36,38,39]. Finally, hexadecanal has been seldom identified in *Citrus* species such as *C. reticulata,* providing a wood aroma [37].

Previous comparison studies on *Citrus* rind EO have shown that it constitutes a meaningful metabolite pool containing valuable information with the ability to discriminate between different *Citrus* species. Classification of species based on the EO profile also was revealed to be largely in accordance with their phylogenetic relationship based on genomic similarity [7]. The current study revealed that EO composition has also the potential to be used to discriminate within the same *Citrus* species, as it has shown the potential to differentiate between two different varieties even as extraordinarily similar as those objects of this study, which previous attempts based on morphological characters or less exhaustive metabolomic features were unable to discriminate.

### 2.5. Antioxidant Activity of NL and PO EO

**Figure 4:**
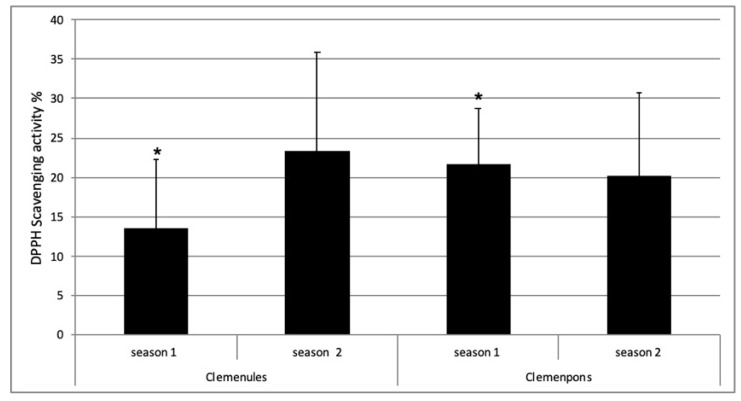
Antioxidant activity of Clemenules (NL) and Clemenpons (PO) EO during two seasons. No statistically significant differences were observed between the two varieties including all data of both seasons and including only data of s2 (*p* > 0.05, according to Student’s *t*-test). * There were statistically significant differences between NL and PO for s1 (*p* < 0.05, according to Student’s *t*-test).

One of the most incipient and leading EO uses is as a good substitute for chemical antioxidants in processing food, preventing lipid oxidation and microorganism growth. The *Citrus* EOs more applied for food packaging and preservation until now are bergamot, lime, and lemon EO and also some tangerines EO [12]. We decided to analyze if both NL and PO EOs showed antioxidant activities, which could enhance their use in the food packaging manufacture. It was also interesting to study whether the specific differences identified between NL and PO would have an effect on their antioxidant activity. Results revealed that all the NL and PO EO analyzed showed antioxidant activity (Figure 4). This result is in accordance with other antioxidant studies of several *Citrus* EO, such as *C. aurantium* [40,41], *C. aurantifolia* [41], *C. reticulata* [8,40], *C. sinensis* [40], *C. limon* [40], *C. lumia* [42], and other *Citrus* species [43,44,45]. In our study, the values of antioxidant activity ranged from 5.03% ± 3.76 to 39.43% ± 5.39 for NL, and from 12.10% ± 4.99 to 31.54% ± 3.04 for PO, including all dates of the two seasons. These data are within the range of those previously obtained for *C. reticulata* and *C. sinensis* using the same method [40].

In s1, PO EO showed more antioxidant activity than NL EO with statistically significant differences, corresponding to one location (Figure 4). However, this result was not confirmed in s2, with two locations, without statistically significant differences between both varieties. Additionally, no statistically significant differences were found between all PO and NL EO antioxidant activities, including the two seasons’ data (Figure 4). Therefore, these results indicate that the differences identified in the chemical composition of these two EOs are not sufficient to influence their antioxidant capacity. Harvest time did not affect antioxidant activity either, as no significant differences were detected along the 2-month collection period for the same variety. These results may be due to the fact that antioxidant activity in Citrus EO is mainly determined by the abundance of monoterpene hydrocarbons [42], although EO bioactivities result from a complex interaction between its constituents, producing both synergistic and antagonistic responses [46]. In the case of the varieties studied, the prevalent compound is the monoterpene hydrocarbon limonene with an abundance of 94–95% [17], and their levels remained constant along the period studied. As far as we know, biological activities of *C. clementina* flavedo EO only have been reported in a few studies. In one of them, antioxidant activities of three *Citrus* EOs, clementine, mandarin (*C. reticulata*), and wilking (*C. reticulata* cultivar wilking), were compared, showing mandarin EO with the highest antioxidant activity and being very similar for clementine and wilking [47]. When VOCs’ composition of these three *Citrus* EOs were compared, it could appreciate that the limonene percentage in clementine and wilking EO were very similar (96–97%), whereas mandarin showed a lower limonene abundance (77%) and a higher γ-terpinene accumulation (15%). These results suggest than when limonene levels are close to 95%, EO antioxidant activities are similar, reducing the role of other minor compounds in this activity. The presence of levels higher than 10% for other monoterpenes such as γ-terpinene or linalool seem to increase the antioxidant activity of these species [8,40,47].

Establishing the relationship between Eos’ composition and their antioxidant activity is crucial for food packaging and preservation aspects in different food matrices [12]. The facts that the antioxidant activity of NL and PO EO did not show significant differences and that their composition was quantitatively very analogous suggest that both EOs can be equally used as a potential source of natural preservative to prevent food oxidation [46,48]. Currently, the efficacy of orange and mandarin peel EOs have already been verified to increase the shelf life of several foods, such as fish, crustaceans, and vegetables [12,49,50,51]. The antioxidant properties of these EOs, along with their antimicrobial properties described in many studies [11,46,47,48], promotes serving EO extracted from *Citrus* peel residues of juice processing industries as natural and safe compounds to prevent food degradation and to prolong its shelf life. Thus, this use of *Citrus* EO contributes to the sustainable development of the juice processing and food preservation industries.

## 3. Materials and Methods

### 3.1. Plant Material

In s1, fruits were collected from NL and PO trees (*C. clementina*), grafted on Carrizo citrange rootstock, located on P5 of the experimental station of the Valencian Institute of Agricultural Research (IVIA, Moncada, Valencia), which has a Mediterranean climate. In s2, fruits were collected from P5 and additionally also from another plot (GCP) of the same station. The total rainfall was 429 mm in s1 and 391 mm in s2 and the average temperature between October and December was 14.8 °C and 12.5 °C in s1 and s2, respectively. Trees of both varieties were grown under the same soil, environmental conditions, agricultural practice, and orientation. 

### 3.2. Sampling and EO Extraction

In both seasons, NL and PO fruits were collected on a weekly basis along almost two months, from October to December (seven harvest days for season, variety, and location, except for NL in s1, which was eight harvest days). Fifteen fruits from three trees were taken every collection day per variety and per location, in an optimal state of ripeness and without any injury. Two EO extractions were planned from these 15 fruits, using only five fruits randomly selected for each extraction. For that, about 50 g of rinds from these five fruits were obtained, whose albedo was manually removed. The resulting fresh flavedo was stored at −80 °C until the EO extraction was carried out within six days.

Flavedo from all 86 samples was thawed at room temperature for 10 min and then placed in a 250-mL round flask with 120 mL of distilled water. This flask was coupled to a Clevenger distillation system that was heated at 80 °C for 3 h. After this period, oil sample was collected, usually 0.5 mL in volume. The yield of all extractions ranged between 1.0–1.1% (mL/100 g of fresh flavedo weight). These samples were kept frozen at –20 °C until analysis. A total of 86 analyses were performed.

### 3.3. Analysis of the EO by GC-MS after Head Space-Solid Phase Microextraction (HS-SPME) 

The EO were initially diluted 1:100 in dichloromethane, and 10 µL of this solution was placed in a 10-mL vial with screw cap and diluted with 990 µL of milli-Q water, with the final EO concentration being 100 nL/mL (100 ppm). VOCs were captured by HS-SPME from the vial head space (HS). For this, vials were first incubated at 50 °C for 10 min under constant agitation at 500 rpm. A 65-μm PDMS/DVB fiber was then introduced into the vial HS for 20 min, under the same conditions of temperature and agitation. Finally, VOCs adsorbed on the fiber were desorbed at 250 °C for 1 min in splitless mode in the injection port of a 6890 N Gas Chromatograph, coupled to a 5975B Mass Spectrometer (Agilent) equipped with a CombiPAL autosampler (CTC Analytics). After fiber desorption, it was introduced into a conditioning station at 250 °C for another 5 min in order to avoid cross contamination. For GC, a DB-5ms capillary column (60 m length, 0.25 mm internal diameter, 1 μm stationary phase) (J&W) was used. Oven ramp was 40 °C for 2 min, then the temperature was increased 5 °C/min until 250 °C, then maintained at 250 °C for 5 min. Helium flow was 1.2 mL/min. Detection was performed by mass spectrometry, with 70 eV electron impact ionization. GC interface and MS source temperatures were 260 °C and 230 °C, respectively. Acquisition was carried out in scan mode in the *m/z* range 35 to 220 (seven scans per second). Chromatograms and mass spectra were recorded using the Enhanced ChemStation E.02.02 software (Agilent).

### 3.4. VOCs’ Identification and Quantification

Differential compounds were unequivocally identified by comparison of both mass spectra and retention times with those of pure standards, with the only exception of tetradecanal, hexadecanal, *p*-menth-1-en-9-al, and β-sinensal, which were tentatively identified based on comparison of mass spectrum and Linear Retention Index (LRI) similarity with those in the NIST05 Mass Spectral Library. Standards were supplied by Sigma-Aldrich (Spain) and Extrasynthese (France). In the cases when mass spectrum similarity was not high enough to be reliable, the compound was labeled as unidentified, and a tentative chemical structure was proposed based on its mass spectrum.

For compound quantification, a specific ion (*m/z*) was chosen for each compound based on the highest signal-to-noise ratio, and the corresponding peak area was integrated.

### 3.5. Antioxidant Activity

Antioxidant capacity was evaluated by measuring spectrophotometrically at 517 nm the ability of quench of the radical 2,2-diphenyl-1-picrylhydrazyl (DPPH) [52]. An aliquot of 50 μL of EO was added to 750 μL of ethanol 96° and 250 μL of ethanolic solution containing DPPH 0.5 mmol/L. The blank sample was prepared using 800 μL of ethanol and 250 μL of the same DPPH solution in order to check the radical stability. After incubation of the mixture at 25 °C for 10 min in the dark, the absorbance at 517 nm was measured using a Pharmacia Biotech 1000E UV-vis spectrophotometer. The radical scavenging activity (S) was expressed in percentage and calculated as S = 100 − [(Ax/A0) × 100], where Ax is the optical density of DPPH solution in the presence of EO and A0 the optical density of DPPH solution in its absence. The antioxidant activity of 86 EO was analyzed (42 PO EO and 44 NL EO) using three technical replicates.

### 3.6. Statistical Analysis

The GC-MS data were processed with the MetAlign software (WUR-PRI) for the alignment of the chromatograms and the quantitation of each MS feature. The resulting data set was submitted to PCA and PLS-DA by the SIMCA-P software v. 11.0 (Umetrics, Umeå, Sweden) using unit variance (UV) and Pareto scaling, respectively. In both analyses, the ratio between levels of each metabolite detected in each sample to their levels in the reference sample were log 2 transformed. The statistical differences (*p*-value < 0.05) between two groups were calculated by means of Student’s *t*-test. Analysis of variance (ANOVA) was performed when comparing more than two groups using the IBM SPSS v.19 Package.

## 4. Conclusions

The present study revealed that, although no differences were identified in the qualitative composition of PO and NL EO, the untargeted metabolomics approach performed followed by multivariate data analysis allowed the identification of specific VOCs differentially accumulated between these two *Citrus* varieties, although they are very closely related and morphologically identical. The differential metabolites identified discriminated between PO and NL consistently even in two different seasons and locations and all along the 2-month harvest period, thus indicating their potential as metabolic markers to differentiate between these two varieties.

The rind essential oil of PO showed higher accumulation of several specific monoterpenes and sesquiterpenes than NL. At the same time, NL showed higher levels of aldehyde and alcohol single-chain aliphatic non-terpenoid compounds, which are associated with the catabolism of fatty acids. Therefore, it seems that the somatic mutation, which originated PO from NL, involved a reduction in specific pathways of fatty acid metabolism together with an enhanced biosynthesis of some specific terpenoids.

A minor but consistent evolution was observed in the EO volatile profile along the harvest time, which was markedly parallel between both varieties and did not interfere with the metabolites identified as discriminant variety markers.

Antioxidant activity was similar in both varieties and, as previously observed for other characters previously studied, it was not able to discriminate between NL and PO.

The discriminant volatile profile of PO as compared to NL, showing higher levels of specific monoterpenoids and lower accumulation of several linear aldehydes and alcohols, could provide distinctive olfactory nuances (more towards caraway in the case of PO and more towards fat for NL), and could be responsible of the distinctive PO aroma previously reported. Nevertheless, it would be necessary to carry out a complementary study using GC-olfactometry, since the human nose does not have to appreciate necessarily the concentration variations of VOCs determined using GC-MS. However, the capability to differentiate the fruits of two varieties as similar as NL and PO based on the analysis of their EO confirms that this is a powerful technique to differentiate between *Citrus* varieties that are morphologically indistinguishable.

## Figures and Tables

**Table 1:** Volatile Organic Compounds more abundant in Clemenpons (PO) than in Clemenules (NL).

Compound	LRI ^a^	Compound Type	Smell [26]	Ratio PO/NL ^b^
3-carene	1024	MTH	lemon	1.44 ± 0.36 ^b^
α-terpinene	1030	MTH	lemon	1.26 ± 0.29 ^b^
γ-terpinene	1070	MTH	turpentine	1.43 ± 0.39 ^b^
unidentified	1138	OMT	-	2.79 ± 1.89 ^b^
unidentified	1161	OMT	-	3.32 ± 2.79 ^b^
*trans*-carveol	1238	OMT	caraway (anise)	2.11 ± 0.79 ^b^
*p*-menth-1-en-9-al ^c^	1242	OMT	-	1.91 ± 0.80 ^b^
unidentified	1311	OMT	-	2.03 ± 0.97 ^b^
α-terpinyl acetate	1363	OMT	wax	1.68 ± 0.51 ^b^
unidentified	1423	OMT	-	1.71 ± 0.55 ^b^
unidentified	1498	STH	-	1.13 ± 0.15 ^b^
α-humulene	1502	STH	wood	1.12 ± 0.18 ^b^
unidentified	1529	STH	-	1.62 ± 0.49 ^b^
unidentified	1548	STH	-	1.43 ± 0.41 ^b^
β-sinensal ^c^	1712	OST	sweet	1.83 ± 0.45 ^b^

^a^ LRI= Linear Retention Index. ^b^ Abundance means for each compound of this table showed statistically significant differences between both varieties, *p* < 0.05, according to Student’s *t*-test. ^c^ Compound tentatively identified. MTH = Monoterpene Hydrocarbon; OMT = Oxygenated Monoterpene; STH = Sesquiterpene Hydrocarbon; OST = Oxygenated sesquiterpene.

**Table 2:** Volatile Organic Compounds more abundant in Clemenules (NL) than in Clemenpons (PO).

Compound	LRI ^a^ (Min)	Compound Type	Smell [26,36,37,38]	Ratio PO/NL ^b^
1-octanol	1071	alcohol	moss, mushroom	0.91± 0.32 ^b^
1-decanol	1275	alcohol	fatty	0.83 ± 0.38 ^b^
dodecanal	1415	aldehyde	fatty, waxy	0.72 ± 0.22 ^b^
tetradecanal ^c^	1620	aldehyde	fatty, wood, waxy, floral	0.61 ± 0.19 ^b^
hexadecanal ^c^	1823	aldehyde	wood	0.65 ± 0.31 ^b^

^a^ LRI = Linear retention index. ^b^ Abundance means for each compound of this table showed statistically significant differences between both varieties, *p* < 0.05, according to Student’s *t*-test. ^c^ Compound tentatively identified.

## Supplementary Materials

The following are available online at https://www.mdpi.com/article/10.3390/plants10091789/s1. Figure S1: Total Ion Count GC-MS chromatogram of a Clemenpons rind essential oil sample. Figure S2: Score plot of the Principal Component Analysis (PCA) based on the whole array of the mass spectra within a *m*/*z* range from 35 to 220 obtained from samples of Clemenules (NL: green) and Clemenpons (PO: blue) collected during season 2; numbers 1 to 7 account for harvest day, from earlier date (1) to later date (7). PC1: first principal component; PC2: second principal component. Table S1: Volatile compounds showing a statistically significant evolution during the harvesting period.
