# Supplementary material for: Untargeted Metabolomics of Rind Essential Oils Allowed to Differentiate Two Closely Related Clementine Varieties
Source: Plants (Basel). 2021 Aug 27;10(9):1789. doi: 10.3390/plants10091789 (PMC8470288; doi:10.3390/plants10091789)
Supplement: Supplementary file 1 [file plants-10-01789-s001.zip › Figure S2_SuppInfo.pdf]

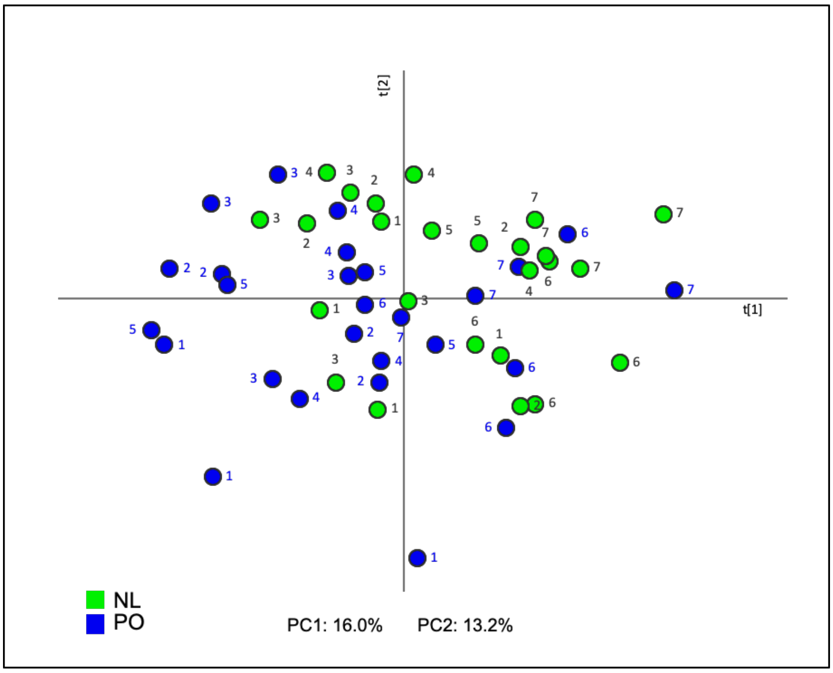

Figure S2. Score plot of the Principal Component Analysis (PCA) based on the whole array of the mass spectra within a *m*/*z* range from 35 to 220 obtained from samples of Clemenules (NL: green) and Clemenpons (PO: blue) collected during season 2, numbers 1 to 7 account for harvest day, from earlier date (1) to later date (7). PC1: first principal component; PC2: second principal component.
