# Supplementary material for: Untargeted Metabolomics of Rind Essential Oils Allowed to Differentiate Two Closely Related Clementine Varieties
Source: Plants (Basel). 2021 Aug 27;10(9):1789. doi: 10.3390/plants10091789 (PMC8470288; doi:10.3390/plants10091789)
Supplement: Supplementary file 1 [file plants-10-01789-s001.zip › Table_S1_SuppInfo .pdf]

Table S1. Volatile organic compounds showing a statistically significant evolution during the harvesting period.

LRI	Type of compound	Probable Formula	Mass spectrum similarity	MS similarity	Season 1 Ratio 6-8/1-3	Season 1 p-value	Season 2 Ratio 6-7/1-3	Season 2 p-value
936	MTH	C10H16			0,70	<0.01	0,56	<0.01
948	MTH	C10H16	α-pinene	STD	0,82	<0.05		ns
986	MTH	C10H16	β-phellandrene	913, 929	0,66	<0.05	0,70	<0.01
992	MTH	C10H16	β-myrcene	STD	0,89	<0.05	0,61	<0.01
997	MTH	C10H16	β-pinene	STD	0,68	<0.01	0,67	<0.01
1070	MTH	C10H16	γ-terpinene	STD	0,73	<0.01	0,64	<0.01
1158	MTAld	C10H18O	citronellal	STD	1,40	<0.01		ns
1270	Fatty acid derivative	C10H18O	(*E*)-2-decen-1-ol	901, 950	1,70	<0.01	2,01	<0.01
1275	Fatty acid derivative	C10H22O	1-decanol	STD	0,61	<0.01	0,57	<0.01
1304	MTAld	C10H18O		850, 882	1,50	<0.01		ns
1331	Fatty acid derivative	C10H16O	(*E*,*E*)-2,4-decadienal	STD	1,80	<0.01		ns
1376	STH	C15H24	α-cubebene	917, 937		ns	0,74	<0.01
1402	Fatty acid derivative	C12H22O	dodecenal	846, 821	1,56	<0.01	1,32	<0.01
1417	STH	C15H24			0,73	<0.01	0,73	<0.01
1423	Oxygenated terpenoid				1,76	<0.01	1,71	<0.01
1459	STH	C15H24		920, 923	0,62	<0.01	0,56	<0.01
1464	STH	C15H24	β-caryophyllene	STD	0,78	<0.01	0,80	<0.01
1511	STH	C15H24	α-farnesene	STD	0,73	<0.05	0,62	<0.01
1547	STH	C15H24		868, 875	0,62	<0.01	0,74	<0.01
1569	STH	C15H24		853, 858	0,71	<0.01	0,49	<0.01
1572	STH	C15H24		837, 870	0,76	<0.01	0,69	<0.01
1596	Fatty acid derivative	C16H28O	hexadecadienal	788, 866	1,85	<0.01	1,76	<0.01
1683	STOH	C15H24O			1,40	<0.05	1,18	<0.01
1712	STAld	C15H22O	β-sinensal	861, 909	0,71	<0.01	0,67	<0.01
1827	Fatty acid derivative	C18H34O	octadecenal	723, 725	1,58	<0.05		ns

Unequivocal identification with a pure standard is indicated as STD. When it was not available, a tentative identification was performed by mass spectral similarity and Linear Retention Index. MTH, Monoterpene Hydrocarbon; MTAld, Monoterpene Aldehyde; STH, Sesquiterpene Hydrocarbon; STOH, Sesquiterpene Alcohol; STAld, Sesquiterpene Aldehyde. MS similarity corresponds to the direct and reverse match of mass spectrum when compared to the NIST05 Mass Spectral Library. A tentative identity has been asigned to those compounds with higher mass spectral similitude based on MS and Linear Retention Index.
