# Supplementary figures and images for: Untargeted Metabolomics of Rind Essential Oils Allowed to Differentiate Two Closely Related Clementine Varieties
Source: Plants (Basel). 2021 Aug 27;10(9):1789. doi: 10.3390/plants10091789 (PMC8470288; doi:10.3390/plants10091789)

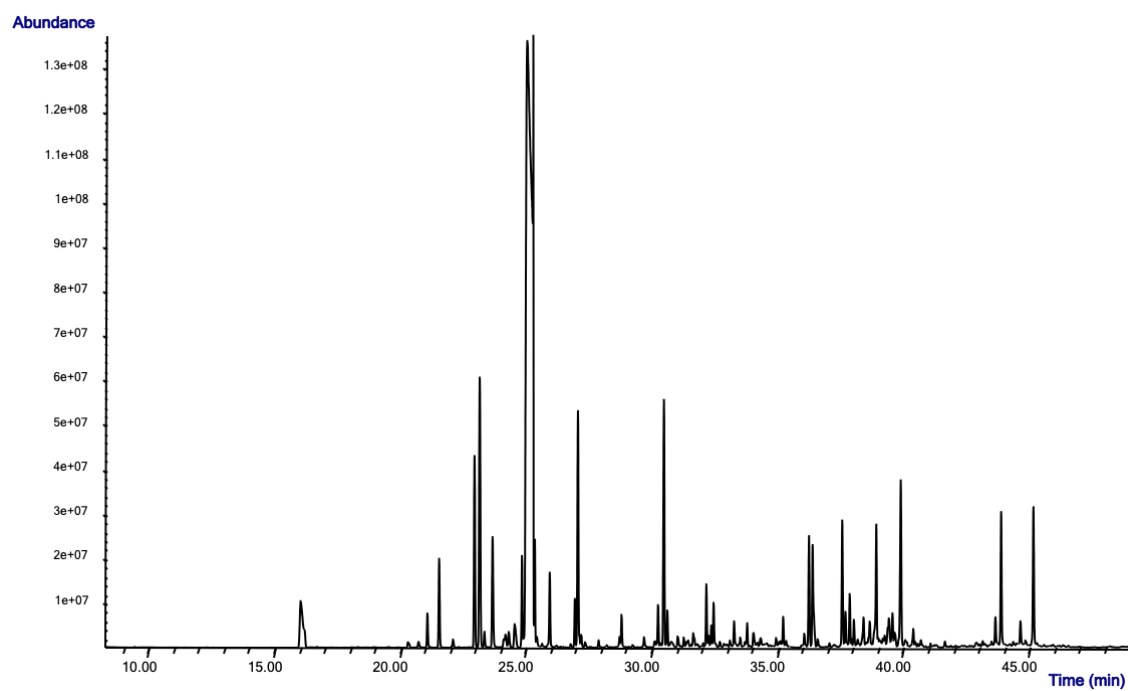

**Figure S1.** Total Ion Count GC-MS chromatogram of a Clematis rind essential oil sample.

Supplement: Supplementary file 1 [file plants-10-01789-s001.zip › Figure S1_SuppInfo.pdf]
